# Absence of ERK5/MAPK7 delays tumorigenesis in *Atm*^−/−^ mice

**DOI:** 10.18632/oncotarget.12908

**Published:** 2016-10-25

**Authors:** Alba Granados-Jaén, Maria Angulo-Ibáñez, Xavier Rovira-Clavé, Celina Paola Vasquez Gamez, Francesc X. Soriano, Manuel Reina, Enric Espel

**Affiliations:** ^1^ Celltec-UB, Department of Cell Biology, Physiology and Immunology, Faculty of Biology, University of Barcelona, Barcelona, Spain; ^2^ Servei d'Anatomia Patològica, Hospital de la Santa Creu i Sant Pau, Barcelona, Spain

**Keywords:** thymic lymphoma, DNA damage response, γH2AX, BMK1, thymocyte

## Abstract

Ataxia-telangiectasia mutated (ATM) is a cell cycle checkpoint kinase that upon activation by DNA damage leads to cell cycle arrest and DNA repair or apoptosis. The absence of *Atm* or the occurrence of loss-of-function mutations in *Atm* predisposes to tumorigenesis. MAPK7 has been implicated in numerous types of cancer with pro-survival and pro-growth roles in tumor cells, but its functional relation with tumor suppressors is not clear. In this study, we show that absence of MAPK7 delays death due to spontaneous tumor development in *Atm*^−/−^ mice. Compared with *Atm*^−/−^ thymocytes, *Mapk7*^−/−^*Atm*^−/−^ thymocytes exhibited an improved response to DNA damage (increased phosphorylation of H2AX) and a restored apoptotic response after treatment of mice with ionizing radiation. These findings define an antagonistic function of ATM and MAPK7 in the thymocyte response to DNA damage, and suggest that the lack of MAPK7 inhibits thymic lymphoma growth in *Atm*^−/−^ mice by partially restoring the DNA damage response in thymocytes.

## INTRODUCTION

ATM is a serine/threonine protein kinase that is rapidly activated by several stressors including nuclear replication stress [1], oxidative stress [2] [3] and mitochondrial dysfunction [4] [5]. Activation of ATM by DNA damage triggers cell cycle checkpoints, including G1/S and G2/M [6] [7], and DNA repair or apoptosis, preventing the transmission of mutations to daughter cells. The absence of ATM in humans and mice increases the incidence of tumors of predominantly lymphoid origin, with most tumors appearing in *Atm*^−/−^ mice being thymic lymphomas [8] [9].

MAPK7 (also named ERK5) is a member of the mammalian mitogen-activated protein kinase family with a kinase domain homologous to that of MAPK3 (ERK1) and MAPK1 (ERK2), and a unique C-terminal domain that determines its cellular location and activity [10]. Deletion of *Mapk7* in mice leads to embryonic lethality due to defective vascular and cardiac development [11–13]. MAPK7 controls the expression of genes that regulate angiogenesis and cardiovascular development [14], is necessary for endothelial homeostasis [15] [16] and contributes to B-cell survival [17] and erythropoiesis in mice [18].

MAPK7 is deregulated in numerous types of cancer [19], including lymphoma [20]. MAPK7 may also mediate chronic inflammation-dependent tumorigenesis in the skin [21] [22]. The tumorigenicity of MAPK7 has been related to its capacity to interact with promyelocytic leukemia protein and inhibit its tumor suppressor activity [23]. By blocking the interaction of promyelocytic leukemia protein with MDM2, active MAPK7 might facilitate the inhibition of tumor suppressor p53 by MDM2 [24]. Based on the use of the MAPK7 inhibitor XMD8-92, it has been shown that MAPK7-dependent inhibition of p53 contributes to tumor growth in a xenograft model [23] [24], though XMD8-92 could have another cellular target besides MAPK7 [25].

In contrast to the role of ATM in triggering cell cycle checkpoints upon DNA damage, MAPK7 activity peaks at G2/M and is required for progression through this cell cycle phase [26]. The data describing opposing roles of ATM and MAPK7 in cell cycle regulation and tumor growth suggest that these could be functionally linked. To test this hypothesis, and as *Atm*^−/−^ mice show a strong predisposition to thymic lymphomas, we eliminated MAPK7 in the hematopoietic system of *Atm*^−/−^ mice and assessed the time of tumor appearance. This approach can yield information on the role played by MAPK7 during the spontaneous progression of a cell toward malignancy. We show that loss of *Mapk7* in the hematopoietic system of *Atm*^−/−^ mice increases the DNA damage response in thymocytes and delays death by spontaneous tumor development in *Atm*^−/−^ mice.

## RESULTS

### Absence of MAPK7 in the hematopoietic system delays death by spontaneous tumor development in *Atm*^−/−^ mice

To determine whether *Mapk7* and *Atm* are functionally linked in tumorigenesis, and given that *Atm*^−/−^ mice spontaneously develop thymic lymphomas, we removed *Mapk7* in the hematopoietic system of *Atm*^−/−^ mice, and assessed the spontaneous rate of tumor development in *Atm*^−/−^ and in *Atm/Mapk7*-double null mice. To this end, *Atm*^+/−^ mice were crossed to *Mapk7^loxP/loxP^vav-cre* mice (hereafter referred to as *Mapk7*^hemat−/−^ mice; Supplementary Figure 1), which lack *Mapk7* in hematopoietic cells [27] [28] or to control *Mapk7^loxP/loxP^* mice (referred to as wild-type mice), and double heterozygous F1 progeny were crossed to obtain F2 *Mapk7*^hemat−/−^*Atm*^−/−^ mice and *Mapk7^loxP/loxP^Atm*^−/−^ mice (referred to as *Atm*^−/−^ mice).

Most *Mapk7*^hemat−/−^*Atm*^−/−^ mice grew to adulthood and showed the low body weight characteristic of *Atm*^−/−^ mice (data not shown). Genotyping of young offspring from 11 (*Mapk7*^hemat−/−^*Atm*^+/−^) x (*Mapk7^loxP/loxP^Atm*^+/−^) crosses and 9 *Mapk7*^hemat−/−^*Atm*^+/−^ mice intercrosses revealed no statistical differences in the frequency of mice homozygous for *Atm* (*p* = 0.35 by chi-squared analysis) as compared with the expected Mendelian frequency (Supplementary Figure 2). However, mice with null homozygosis for both *Atm* and *Mapk7* were born at a lower frequency than expected (*p* = 0.024), suggesting that the absence of both genes in the hematopoietic system produces a developmental drawback. However, the possibility that it is the presence of Cre recombinase, rather than the absence of MAPK7 what affects the birth rate of *Atm*^−/−^ mice cannot be excluded.

**Figure 1:**
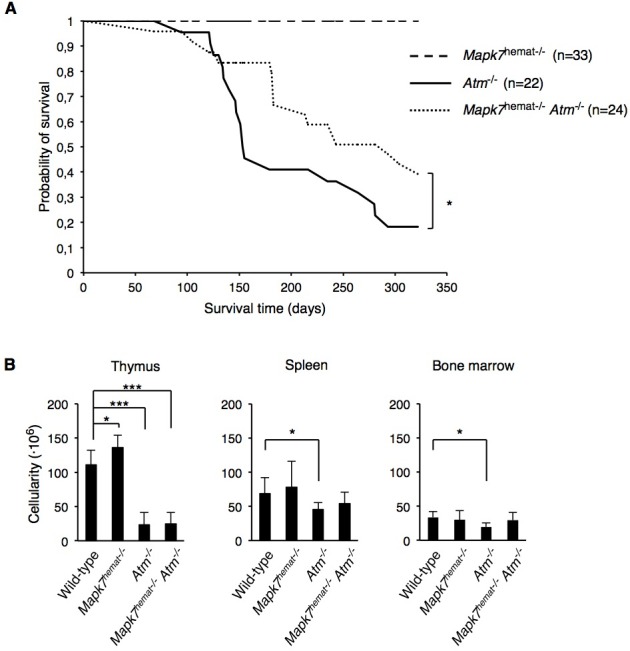
Absence of MAPK7 delays tumorigenesis in *Atm*^**−/−**^ mice **A.** Kaplan-Meier survival curves of *Mapk7*^hemat−/−^ (*n* = 33), *Atm*^−/−^ (*n* = 22) and *Mapk7*^hemat−/−^*Atm*^−/−^ (*n* = 24) mice over 320 days. P values were obtained by the log-rank test. **B.** Total cellularity of wild-type (*n* ≥ 5), *Mapk7*^hemat−/−^ (*n* = 7), *Atm*^−/−^ (*n* = 7) and *Mapk7*^hemat−/−^*Atm*^−/−^ (*n* ≥ 8), in the indicated organs.

We then assessed the survival of these adult mice. In agreement with previous studies, we did not observe spontaneous tumor formation in *Mapk7*^hemat−/−^ mice [17] [18] [27] [29] and no mice died during the first year of life (Figure 1A). In contrast, the median survival time of *Atm*^−/−^ mice was 153 days of age (Figure 1A) with ~80% of these mice having died at 300 days of age. Interestingly, the median survival time of *Mapk7*^hemat−/−^*Atm*^−/−^ mice was 281 days of age (Figure 1A). Thus, *Mapk7*^hemat−/−^*Atm*^−/−^ mice showed a significant increase in survival compared to *Atm*^−/−^ mice (*p* = 0.02 at day 320, log-rank test), indicating that MAPK7 deficiency in the hematopoietic system delays death by spontaneous tumor development in *Atm*^−/−^ mice and suggesting an antagonistic interaction between the MAPK7- and ATM-signaling pathways affecting thymic lymphoma development.

Most tumors appearing in *Atm*^−/−^ and *Mapk7*^hemat−/−^*Atm*^−/−^ mice were thymic lymphomas. However, older mice (~1 year or more) presented tumors of different types or died of unknown causes associated with a wasting syndrome (Supplementary Table I). The longer survival of *Mapk7*^hemat−/−^*Atm*^−/−^ mice might facilitate the emergence of other disorders linked to the absence of ATM [30]. The thymic tumor cells were clonal, showed either an immature phenotype with a CD4^+^CD8^+^ signature or were single-positive (SP) thymocytes (Supplementary Figure 3).

### Normal erythropoiesis in *Mapk7*^hemat−/−^*Atm*^−/−^ mice and impaired B cell development in *Atm*^−/−^ and *Mapk7*^hemat−/−^*Atm*^−/−^mice

Some hematopoietic-specific traits of *Atm*^−/−^ mice, such as reduced thymocyte cellularity, were also evident in *Mapk7*^hemat−/−^*Atm*^−/−^ mice, whereas *Mapk7*^hemat−/−^ mice presented a small increase (Figure 1B). Interestingly, *Atm*^−/−^ mice, but not *Mapk7*^hemat−/−^*Atm*^−/−^ mice, showed also reduced cellularity in the bone marrow and spleen. Absence of *Atm* or *Mapk7* leads to hematopoietic impairment [17] [18] [30], we therefore analyzed in more detail the erythroid and B cell development in the bone marrow in 4- to 8-week-old mice.

**Figure 2:**
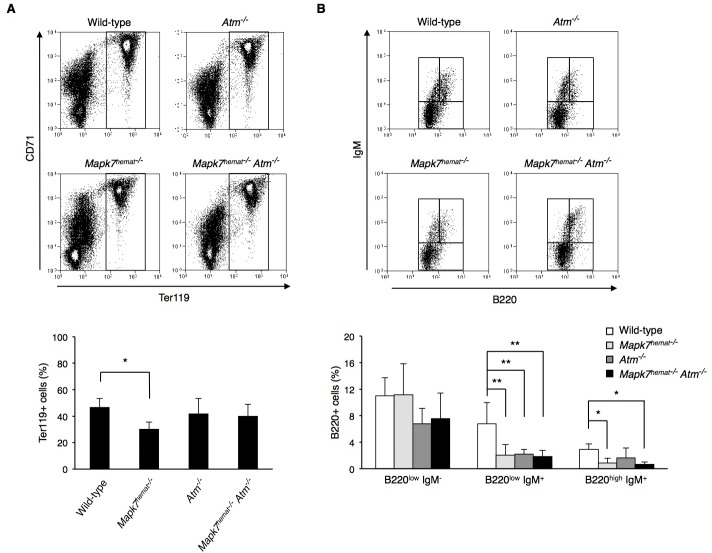
Normal erythropoiesis in *Mapk7*^hemat−/−^*Atm*^−/−^mice and impaired B cell development in *Atm* and *Mapk7*^hemat−/−^ *Atm*^−/−^ mice **A.** Representative flow cytometry profiles representing the erythroid population in the bone marrow. Dot plots representing erythroid precursor distribution based on ter119 and CD71 expression (top). Percentage of ter119^+^ population in wild-type (*n* = 3), *Mapk7*^hemat−/−^ (*n* = 7), *Atm*^−/−^ (*n* = 3) and *Mapk7*^hemat−/−^*Atm*^−/−^ (*n* = 6) bone marrow (bottom). **B.** Representative flow cytometry profiles representing B cell precursor distribution (B220^low^IgM^−^, B220^low^IgM^+^, B220^high^IgM^+^) in the bone marrow population (top). Percentage of bone marrow B cell precursors in wild-type (*n* = 3), *Mapk7*^hemat−/−^ (*n* = 7), *Atm*^−/−^ (*n* = 3) and *Mapk7*^hemat−/−^*Atm*^−/−^ (*n* = 6) mice.

The generation of erythropoietic precursors (cells CD71^+^ter119^+^) diminished in the bone marrow of *Mapk7*^hemat−/−^ mice but not in *Atm*^−/−^ mice (Figure 2A) [18]. Interestingly, the erythropoiesis impairment observed in *Mapk7*^hemat−/−^ mice disappeared in *Mapk7*^hemat−/−^*Atm*^−/−^ mice, indicating that the absence of *Atm* compensates for the absence of *Mapk7* during erythropoiesis in bone marrow of young mice (Figure 2A, bottom).

The analysis of B cell progenitors in the bone marrow showed that generation of the B cell precursor B220^low^IgM^+^ was impaired in the three genotypes: *Mapk7*^hemat−/−^, *Atm*^−/−^ and *Mapk7*^hemat−/−^*Atm*^−/−^ mice (Figure 2B) [17] [18]. Moreover, the generation of the B cell precursor B220^high^IgM^+^ was also impaired in *Mapk7*^hemat−/−^ and *Mapk7*^hemat−/−^*Atm*^−/−^ mice. The combined absence of *Mapk7* and *Atm* did not have an additive effect on decreasing further the bone marrow B220^low^IgM^+^ or B220^high^IgM^+^ pools.

In the lymph nodes and spleen, the percentage of mature B cells was not affected by the absence of *Atm* and/or *Mapk7.* Regarding T cells, while CD8^+^ T cells were not altered, a decrease in the percentage of CD4^+^ T cells was observed in the lymph nodes of *Atm^−/−^* mice and in the spleen of mice lacking both *Mapk7* and *Atm* (Supplementary Figure 4).

These results indicate that these two kinases play a different role in erythroid and B cell development, with both kinases contributing to B cell development, but only *Mapk7* being necessary for normal erythroid development in young mice.

### Equivalent thymic cellular distribution in *Atm*^−/−^ and *Mapk7*^hemat−/−^*Atm*^−/−^ mice

**Figure 3:**
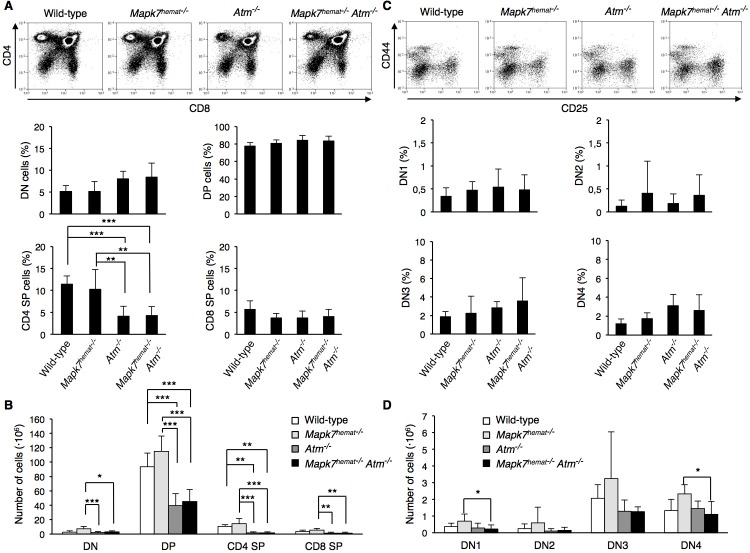
Equivalent thymic cellular distribution in *Atm*^−/−^ and *Mapk7*^hemat−/−^*Atm*^**−/−**^ mice **A.** Representative flow cytometry profiles representing thymic cell distribution based on CD4 and CD8 expression (top). Percentage of thymic cell subsets (bottom). Thymocytes were isolated from 4- or 5-week-old wild-type (*n* = 6), *Mapk7*^hemat−/−^ (*n* = 7), *Atm*^−/−^ (*n* = 7) and *Mapk7*^hemat−/−^ *Atm*^−/−^ (*n* = 8) mice, stained with anti-CD4-APC and anti-CD8-FITC, and then analyzed by flow cytometry. Data represent mean +/− SD of CD4^−^CD8^−^ DN, CD4^+^CD8^+^ DP and SP CD4^+^ and CD8^+^ cell subsets. **B.** Number of thymocytes in each developmental stage, for the same mice analyzed in **A.**. **C.** Representative flow cytometry profiles of thymic DN population based on CD44 and CD25 expression (top). Percentage of DN thymocyte cell subsets (bottom). Thymocytes were isolated from 4- and 5-week-old wild-type (*n* = 6), *Mapk7*^hemat−/−^ (*n* = 7), A*tm*^−/−^ (*n* = 7) and *Mapk7*^hemat−/−^*Atm*^−/−^ (*n* = 8) mice, stained with anti-CD4-APC, anti-CD8-alexa 488, anti-CD44-PerCP and anti-CD25-PE, and then analyzed by flow cytometry. Data represent mean +/− SD of CD44^+^CD25^−^ (DN1), CD44^+^CD25^+^ (DN2), CD44^−^CD25^+^ (DN3) and CD44^−^CD25^−^ (DN4) subsets. **D.** Number of thymocytes in each DN cell subset for the same mice analyzed in **C.**.

We then assessed whether *Mapk7* and *Atm* are functionally linked during T cell development, by performing a flow cytometry analysis on thymocytes isolated from 4- or 5-week-old mice. The percentage of SP CD4^+^ thymocytes decreased in mice strains lacking *Atm*, whereas the percentage of CD4^−^CD8^−^ double-negative (DN), CD4^+^CD8^+^ double-positive (DP) and SP CD8^+^ thymocytes did not change (Figure 3A) [9] [31]. When the absolute cell numbers were considered, the DP and SP CD4^+^ thymocytes decreased in mice strains lacking *Atm* (Figure 3B) [31]. *Atm*^−/−^ mice and *Mapk7*^hemat−/−^*Atm*^−/−^ mice presented a similar number of DN, DP and SP thymocytes (Figure 3B).

Thymocytes at DN stage start the recombination of *V(D)J* gene segments, a process that ends at the DP stage and requires the activity of ATM for proper repair of the DNA double-strand breaks (DSB) produced during DNA recombination [31] [32]. The lower number of DP and mature SP CD4^+^ thymocytes in mice strains lacking *Atm* suggests that a partial arrest of thymocyte maturation during the DN stage is taking place in these mice, probably as a result of the incapacity to resolve the DSB generated during DNA recombination [33].

As the main proliferative population, the DN thymocytes were analyzed in more detail. The DN pool can be fractionated in progressively maturing DN1 to DN4 subsets according to the expression of the IL-2 receptor alpha chain (CD25) and CD44 (Figure 3C, 3D). Thymocyte precursors initiate the recombination of *tcrg, tcrd* and *tcrb* mainly at the CD4^−^CD8^−^CD44^−^CD25^+^ (DN3) stage, and completion of this process and expression of pre-TCR leads to differentiation into CD4^−^CD8^−^CD44^−^CD25^−^ (DN4) cells. Neither the percentage of DN populations nor the number of cells in each DN pool did show differences between wild-type and the other genotypes, or between *Atm*^−/−^ mice and *Mapk7*^hemat−/−^*Atm*^−/−^ mice (Figure 3C, 3D). Only *Mapk7*^hemat−/−^ mice and *Mapk7*^hemat−/−^*Atm*^−/−^ mice did show a different number of CD4^−^CD8^−^CD44^+^CD25^−^ (DN1) and DN4 cells (Figure 3D).

Taken together, these results indicate that deletion of *Mapk7* in *Atm*^−/−^ mice did not change the distribution of major thymic populations.

### Increased G2/M cell cycle phase accumulation in DN thymocytes of *Mapk7*^hemat−/−^*Atm*^−/−^ mice

**Figure 4:**
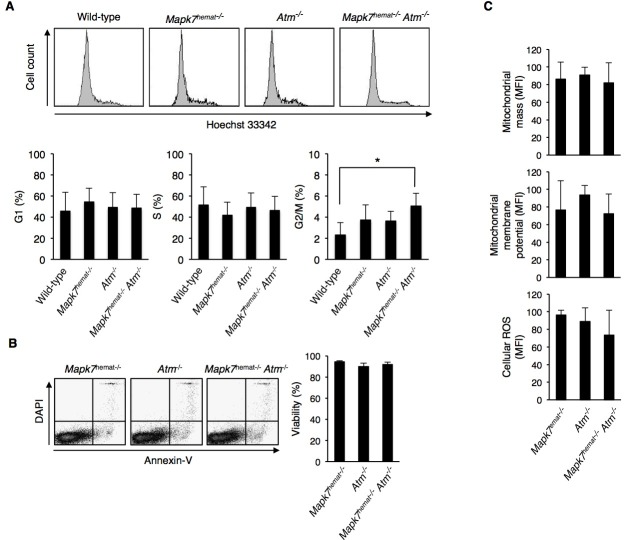
Increased G2/M cell cycle phase accumulation in DN thymocytes of *Mapk7*^hemat−/−^*Atm*^−/−^ mice **A.** Representative flow cytometry profiles of DNA content in DN thymocytes based on Hoechst staining (top). Percentage of DN thymocytes in each cell cycle phase (bottom). Thymocytes were fixed in 1% paraformaldehyde, labeled with anti-CD4 and anti-CD8 antibodies as indicated in Figure 3 and stained with Hoechst 33342. **B.** Percentage of live thymocytes measured immediately after thymic extraction. Representative dot plots (left); quantification of FACS staining (right, *n* ≥ 3 for each genotype). Apoptosis was measured by staining with annexin V and DAPI followed by flow cytometry analysis. **C.** The mitochondrial mass, membrane potential and cellular ROS in thymocytes were determined by staining with mitoTracker-green, mitoTracker-red and dihydrorhodamine-123, respectively, and analyzed by flow cytometry (*n* ≥ 3 for each genotype). The MFI in arbitrary units is shown.

**Figure 5:**
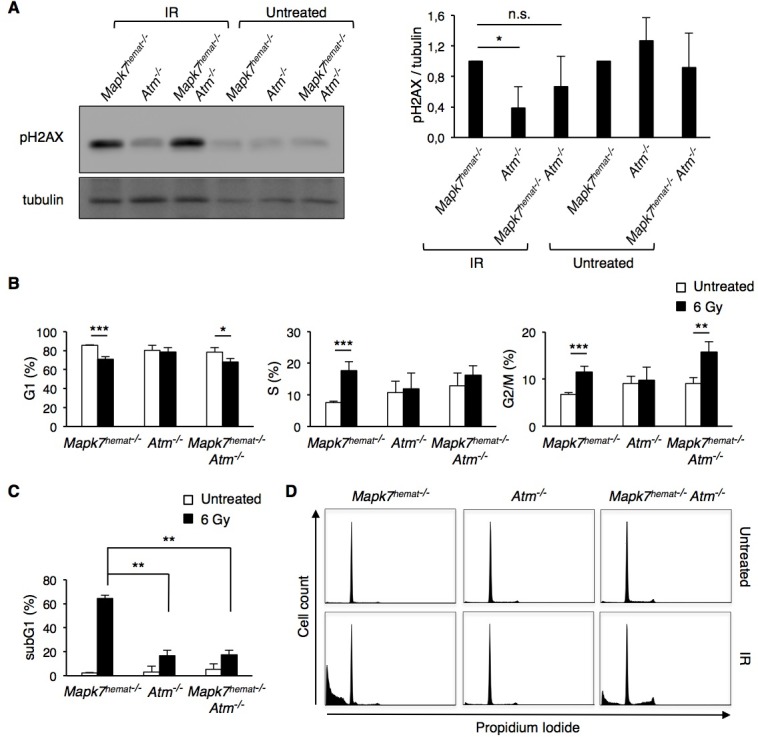
Absence of *Mapk7* in *Atm*^**−/−**^ mice restores the DNA damage response to ionizing irradiation Mice were treated with 6 Gy and two hours later, thymocytes were isolated and lysed in SDS-buffer or fixed for cell cycle analysis. **A.** Phosphorylation of H2AX (pH2AX) in thymocytes isolated from irradiated (IR) mice. Representative Western blot with pH2AX and tubulin as loading control (left); pH2AX:tubulin ratio normalized to control *Mapk7*^hemat−/−^ (right; *n* ≥ 3 for each genotype). n.s., not significant. **B.** Cell cycle distribution of live thymocytes isolated from the same mice evaluated in **A.**, as analyzed by PI staining. **C.** Cell death of the same thymocyte samples evaluated in **B.** (sub-G1 fraction). **D.** Representative flow cytometry profiles of DNA content in thymocytes based on PI staining. The quantitation of these results is represented in **B.** for live thymocytes and in **C.** for dead cells.

Although no major changes between the thymocyte distribution of 4- to 5-week old *Atm*^−/−^ mice and *Mapk7*^hemat−/−^*Atm*^−/−^ mice could be found, the delay in thymic lymphoma development in the latter suggest that the proliferative capacity of thymocytes or their apoptotic response to DNA damage has changed. Cell cycle distribution was assessed in total thymocytes, and a significant increase in G2/M fraction in *Mapk7*^hemat−/−^*Atm*^−/−^ mice was observed in comparison to wild-type mice (Supplementary Figure 5A). As *Atm*^−/−^ thymocytes fail to progress through the DN3 stage [33] and DN thymocytes are the thymic population with proliferative activity, we analyzed the cell cycle distribution of DN thymocytes. Interestingly, DN thymocytes from *Mapk7*^hemat−/−^*Atm*^−/−^ mice presented a higher percentage of cells in the G2/M-phase of the cell cycle (Figure 4A). Besides DN thymocytes, no differences in cell cycle distribution were detected among the different genotypes in DP, CD4^+^ SP and CD8^+^ SP thymocyte subsets (Suplementary Figure 5B), indicating a specific effect of the combined deficiency of *Mapk7* and *Atm* in DN thymocyte proliferation. The increase in the G2/M fraction of DN thymocytes in *Mapk7*^hemat−/−^*Atm*^−/−^ mice did not increase the number of DN cells (Figure 3), suggesting that the lack of *Mapk7* in *Atm^−/−^* DN thymocytes could be inducing a partial G2/M arrest or an increase in thymocyte apoptosis in these mice. We were unable to detect changes in apoptosis, as analyzed by annexin V staining, which was similar in the three genotypes investigated (Figure 4B).

In addition to a deficit in the DNA damage response, the absence of *Atm* leads to mitochondrial dysfunction and oxidative stress [34] [35]. In order to ascertain whether the delay in thymic lymphoma growth observed in *Mapk7*^hemat−/−^*Atm*^−/−^ mice was also related to changes in mitochondrial function, we analyzed the mitochondrial content and membrane potential in thymocytes. No major differences were observed between *Atm*^−/−^ and *Mapk7*^hemat−/−^*Atm*^−/−^ mice in mitochondrial content or membrane potential (Figure 4C). Furthermore and related to the alterations in mitochondria, *Atm*^−/−^ mice of approximately 6 months of age have been described to exhibit an increase in reactive oxygen species (ROS) [30]. Quantitation of ROS in thymocytes of 4- to 5-week old mice by staining with dihydrorhodamine-123 did not show major differences between control *Mapk7*^hemat−/−^, *Atm*^−/−^ and *Mapk7*^hemat−/−^*Atm*^−/−^ mice (Figure 4C). These results suggest that neither differences in mitochondrial activity nor ROS content were responsible for the delay in thymic lymphoma observed in *Mapk7*^hemat−/−^*Atm*^−/−^ mice.

### Absence of *Mapk7*^hemat−/−^*Atm*^−/−^ mice restores the DNA damage response to ionizing radiation

Tumor susceptibility in *Atm*^−/−^ mice is caused by deficiencies in the DNA damage response; therefore, we investigated the effect of *Mapk7* loss on DNA damage signaling. The DSB caused by ionizing radiation activate ATM, which phosphorylates several key participants in the DNA damage response, including histone H2AX, which enables recruitment of DNA repair complexes [36] [37]. Exposure of control *Mapk7*^hemat−/−^ mice to 6-Gy ionizing radiation led to phosphorylation of H2AX, indicating the presence of DNA damage in thymocytes harvested 2 hours after the mice were exposed to radiation (Figure 5A). However, thymocytes isolated from irradiated *Atm*^−/−^ mice showed a deficit in this signaling response (Figure 5A), consistent with published data [38]. Conversely, loss of *Mapk7* in *Atm*^−/−^ mice restored the capacity of thymocytes to respond to DNA damage caused by ionizing radiation, via phosphorylation of H2AX (Figure 5A). In untreated mice, phosphorylation of H2AX in thymocytes presented no differences between the different genotypes. These results indicate that the deficient DNA damage response in *Atm*^−/−^ mice can be restored by loss of *Mapk7*.

Phosphorylation of H2AX promotes the recruitment of checkpoint proteins and initiation of the G2/M checkpoint [39]. We therefore determined the cell cycle phases in thymocytes from irradiated mice. Ionizing radiation did not induce a change in the cell cycle profile of *Atm*^−/−^ thymocytes, indicating radioresistant DNA synthesis (Figure 5B) [40] [41]. Interestingly, thymocytes from irradiated *Mapk7*^hemat−/−^A*tm*^−/−^ mice showed a partial G2/M arrest (Figure 5B). This indicates that in *Atm*^−/−^ thymocytes, the presence of MAPK7 inhibited the G2/M checkpoint triggered by radiation-dependent DNA damage, and this G2/M checkpoint was partially restored in *Mapk7*^hemat−/−^*Atm*^−/−^ thymocytes.

**Figure 6:**
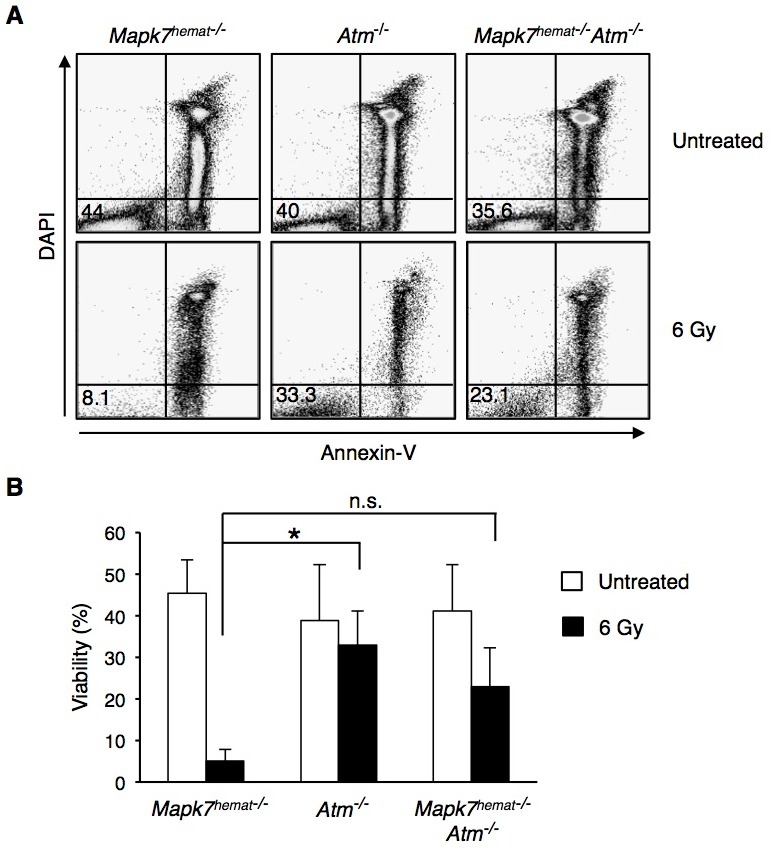
Absence of *Mapk7* in *Atm*^**−/−**^ mice restores the apoptotic response to ionizing irradiation Percentage of live thymocytes after isolation from irradiated mice (6 Gy) and culturing for 20 h. Representative flow cytometry profiles **A.** and quantification of FACS staining (percentage of live cells) **B.**. Cell death was measured by staining with annexin V and DAPI followed by flow cytometry (*n* ≥ 3 for each genotype). n.s., not significant.

As anticipated, irradiation of control *Mapk7*^hemat−/−^ mice provoked an early massive apoptosis of thymocytes (Figure 5C). Although the early apoptotic response of thymocytes from irradiated *Atm*^−/−^ mice and *Mapk7*^hemat−/−^*Atm*^−/−^mice was similar (Figure 5C, 5D), we further examined the apoptosis of these cells after culturing them at 37°C for 20h and staining them with annexin V and DAPI. Whereas the survival of irradiated control *Mapk7*^hemat−/−^ thymocytes was markedly low, a substantial percentage of thymocytes from *Atm*^−/−^ mice survived after mice irradiation and cell culture (Figure 6). Importantly, the loss of *Mapk7* in *Atm*^−/−^ mice partially restored the apoptotic response of thymocytes to DNA damage (Figure 6). Taken together, these results indicate that loss of *Mapk7* in *Atm*^−/−^ mice restores the DNA damage-signaling pathway in thymocytes, leading to G2/M arrest and apoptosis when mice are subjected to irradiation.

### Human cancers with *ATM* mutations do not show increased *Mapk7* mRNA content

The data presented above suggest that in absence of *Atm*, an increase in the expression or function of MAPK7 could be advantageous for oncogenic transformation. To test this hypothesis, we queried The Cancer Genome Atlas (TCGA) clinical database for the expression of *MAPK7* mRNA in human cancers presenting mutated ATM. The results indicate that there is not a statistically significant relationship between the presence of ATM mutations and *MAPK7* mRNA content in human cancer (Supplementary Figure 6).

## DISCUSSION

ATM is activated by DNA damage and is necessary during T cell development to repair DSB generated during *Tcr* recombination [32]. Developing T cells in *Atm*^−/−^ mice present unprotected DNA ends that recombine abnormally [42], leading to immunodeficiency and genetic instability, and ultimately this can provoke T cell lymphoma. However, *Atm*^−/−^ mice lacking *recombinase activating* genes are free of tumors [43].

In this study, we have shown that the loss of *Mapk7* in *Atm*^−/−^ mice delays tumorigenesis, indicating a functional interaction between *Mapk7* and *Atm*. Upon DNA damage, ATM phosphorylates several key participants in the DNA damage response, including H2AX, which enables recruitment of DNA repair complexes [44] and the arrest of DNA replication. In contrast, *Atm*^−/−^ cells show impaired H2AX phosphorylation and radioresistant DNA replication (the present study) [40] [41]. The results from irradiated mice reported here indicate that when *Mapk7* is eliminated in *Atm*^−/−^ mice, H2AX phosphorylation is restored, together with partial G2/M arrest and increased apoptosis of thymocytes. The phosphorylation of H2AX in *Mapk7*^hemat−/−^*Atm*^−/−^ thymocytes is probably responsible for restitution of the G2/M checkpoint [39]. The reported activities of MAPK7 related to cell cycle control include its increased activity at G2/M, facilitating this cell cycle transition [26]. Loss of *Mapk7* might result in stronger activation of the G2/M checkpoint upon DNA damage, as observed in the present study. Restoration of the DNA damage response and the G2/M checkpoint in *Mapk7^hemat^*^−/−^*Atm*^−/−^ thymocytes reduces the generation of DNA instability and probably contributes to the delay of thymic lymphoma in *Mapk7*^hemat−/−^*Atm*^−/−^ mice.

During the *V(D)J* recombination process, ATM, DNA-PKc and H2AX function in a common DNA repair pathway [45]. ATM regulates hairpin-sealed DNA end-processing by phosphorylation of DNA-PKc [46], whereas H2AX has a specific role in preservation of the structural integrity of broken DNA ends during *V(D)J* recombination in murine lymphocytes [47]. The phosphorylation of H2AX in cells under ionizing radiation is mediated by both ATM and DNA-PKc [48]. Therefore, the phosphorylation of H2AX in thymocytes from irradiated *Mapk7*^hemat−/−^*Atm*^−/−^ mice, but not from irradiated *Atm*^−/−^ mice, suggests that in absence of ATM, MAPK7 inhibits DNA-PKc. These data suggest that a normal function of MAPK7, regulated by ATM, may be to inhibit DNA-PKc kinase activity in DN thymocytes, reducing the DNA damage response and apoptosis during *tcr* recombination. If unrestrained by ATM, this MAPK7 activity in developing T cells could therefore lead to genetic instability.

Due to damaged DNA, *Atm^−/−^* thymocytes cannot mature efficiently and their number diminish after the DN stage, showing a shortage of DP and maturing SP CD4^+^ T thymocytes (the present study and [9]). Restitution of the DNA damage response in *Mapk7*^hemat−/−^*Atm*^−/−^ thymocytes did not alleviate arrest at the DN stage, suggesting that although undetected, increased apoptosis of potentially oncogenic DN thymocytes in *Mapk7*^hemat−/−^*Atm*^−/−^ mice contributes to delaying thymic lymphoma development.

We have recently reported that MAPK7 is necessary for normal erythropoiesis in mice, contributing to proper dNTP metabolism in highly proliferative erythroid progenitors [18]. Loss of *Mapk7* in the hematopoietic compartment gives rise to extramedullary erythropoiesis, splenomegaly and an increased mutation rate in erythrocyte precursors, probably due to an imbalance of dNTPs [18]. Contrary to *Mapk7^hemat^*^−/−^ mice, erythropoiesis was restored in the bone marrow of *Mapk7^hemat^*^−/−^*Atm*^−/−^ mice (the present study), indicating that the absence of *Atm* compensates for the absence of *Mapk7* during erythropoiesis in young mice, and suggesting that the erythroid dNTP pool is normalized in *Mapk7^hemat^*^−/−^*Atm*^−/−^ mice. This is consistent with increased cellular dNTP levels during replication stress in *Atm*^−/−^ mice [49].

B cell development in the bone marrow was impaired in the three genotypes studied here, though the spleen and lymph nodes contained a normal number of mature B cells. MAPK7 is necessary in B cells to signal BAFF-surviving stimuli [17], whereas in *Atm*^−/−^ mice the impairment in B cell development is related to a deficient *V(D)J* recombination [50]. Curiously, *Mapk7^hemat^*^−/−^*Atm*^−/−^ mice did not show a stronger alteration of B cell precursors, suggesting that both genes are acting through the same pathway.

In addition to its role in the DDR response, ATM is activated by oxidative stress and is needed to maintain cellular homeostasis in front of oxidative and mitochondrial stressors [2–5] [30]. In the present study, we analyzed whether *Atm*^−/−^ thymocytes presented evidences of oxidative and mitochondrial stress, but no alterations were observed. We hypothesize that a reason why no dysfunctions were detected is that our study on oxidative and mitochondrial activity was carried out with thymocytes from 4- to 5-week-old mice, which were younger than the ones used in other studies [5]. In addition, the genetic background of mice used in the present study could lie behind the apparent mitochondrial normality of *Atm*^−/−^ thymocytes [51].

In an attempt to confirm that the development of cancer in absence of *Atm* is favoured by MAPK7 function, we examined the *MAPK7* mRNA content in human cancers presenting ATM mutations. However, no statistically significant relationship was found between the amount of *MAPK7* mRNA and the presence of mutated ATM. These data do not confirm that MAPK7 provides an advantage to ATM-dependent tumorigenesis, though they do not formally exclude this possibility. MAPK7 function could be beneficial to the oncogenic process when this is initiated by a particular type of ATM mutation. In addition, although we used mRNA data, the mRNA content and the activity of the expressed MAPK7 are distantly related. Therefore, the prospect of using functional data (i.e. MAPK7 activity) will provide a closer view to the MAPK7 - ATM relationship in human cancer.

Taken together, these data establish a functional relationship between *Atm* and *Mapk7* during bone marrow erythropoiesis and suggest a non-redundant role of MAPK7 in thymic precursors during repair of *tcr* recombination-dependent DSB, facilitating mitigation of an excessive DNA damage response and apoptosis. In absence of MAPK7, thymocytes from irradiated *Atm*^−/−^ mice partially recover the G2/M checkpoint, progress with increased difficulty through cell cycle and ultimately show increased apoptosis. These results suggest that MAPK7 inhibitors could be of therapeutic value in lymphoma caused by absence of ATM by re-establishing cell cycle checkpoints in cancer cells.

## MATERIALS AND METHODS

### Ethics statement

Investigation has been conducted in accordance with the ethical standards and according to national and international guidelines and has been approved by the authors' institutional review board (the *Departament d'Agricultura, Ramaderia, Pesca, Alimentació i Medi Natural* of *Generalitat de Catalunya*).

### Mice

*Atm^+l^*^−^ mice were kindly provided by Óscar Fernández-Capetillo (National Cancer Research Centre, Madrid, Spain) [52]. *Mapk7^loxP/loxP^* mice were kindly provided by Cathy Tournier (University of Manchester, Manchester, U.K.) [29]. *Mapk7^loxP/loxP^vav-cre* mice which lack *Mapk7* in hematopoietic cells are described elsewhere [27] [28]. *Mapk7^loxP/loxP^vav-cre* mice were on C57BL/6 background whereas *Atm*^+/−^ mice had a mixed background (129S6, CD-1, C57BL/6)*. Atm*^+/−^ mice were crossed to *Mapk7^loxP/loxP^vav-cre* mice (referred to as *Mapk7*^hemat−/−^mice) or to control *Mapk7^loxP/loxP^* mice (referred to as wild-type mice), and double heterozygous F1 progeny were crossed to obtain F2 *Mapk7*^hemat−/−^*Atm*^−/−^ mice and *Mapk7^loxP/loxP^Atm*^−/−^ mice (referred to as *Atm*^−/−^ mice). The *Mapk7*^hemat−/−^ mice and wild-type mice, used in experiments were F1 crossings, in order to keep the same genetic background for all mice. Experiments were conducted on 4- to 8-week-old mice. To genotype mice, the following oligonucleotides were used: to detect floxed *Mapk7*, forward 5'-tccatgctgttagtcctttgg-3' and reverse 5'-agcggctgtgaagagtgaat-3' (floxed amplicon = 260bp, wild-type amplicon = 200bp). Presence of *Vav-Cre* was analyzed with the oligonucleotides: forward 5'-cgagtgatgaggttcgcaag-3' and reverse 5'-atcttcaggttctgcgggaa-3'. Wild-type *Atm* was detected with oligonucleotides: forward 5'-gctgccatacttgatccatg-3' and reverse 5'- tccgaatttgcaggagttg-3' (wild-type amplicon = 147bp). To detect *neomycin* gene insertion in *Atm* the following oligonucleotides were used: forward 5'-cttgggtggagaggctattc-3' and reverse 5'-aggtgagatgacaggagatc-3' (neomycin amplicon = 280bp).

### Cell culture and reagents

For cell preparations, single cell suspensions were isolated from thymus, spleen and bone marrow of mice. The suspension was counted to determine the organ cellularity, and then mature red blood cells of spleen and bone marrow suspensions were discarded from the suspension by overlaying cells on a solution of 16% iodixanol, 0.63% NaCl and 10 mM Hepes and centrifuged at 900x g for 30 minutes. The mononuclear fraction of the density gradient was collected, washed with PBS, counted and stained or fixed. Cell preparations were cultured in RPMI 1640 medium (ref. BE12-702F, Lonza) supplemented with 9% FBS, penicillin/streptomycin, 2 mM L-glutamine. Mitotracker Green or Red (Life Technologies) was used at a concentration of 50nM for 15 to 30 minutes at 37°C.

### Western blot

Cells were lysed in Laemmli buffer and loaded on 10% or 15% acrylamide gels. Transfer to nitrocellulose membranes and immunoblotting was performed as previously described [18]. Incubation with primary antibody (mouse anti-H2AX phosphorylated S139, Biolegend #613401; rabbit anti-ERK5, Cell Signaling #3372; mouse anti-beta actin, Sigma-Aldrich #A2228; mouse anti-tubulin, Sigma-Aldrich #T9026) was performed overnight at 4°C under agitation. The following day, after washing, the membrane was incubated with the secondary antibody-HRP conjugated (rabbit anti-mouse, Sigma-Aldrich #A9044; goat anti-rabbit, Sigma-Aldrich #A0545) for 1 hour under agitation at room temperature. Western blots were quantified on a Luminescent Image Analyzer LAS-3000 (Fujifilm). Densitometry analysis was performed using ImageJ software.

### Immunostaining and flow cytometry analysis

For extracellular labeling, thymocytes, splenocytes, lymph node cells and bone marrow cells were stained with saturating amounts of primary antibodies conjugated to fluorochromes (, ; anti-CD8 AlexaFluor 488, ; anti-CD44 PerCP, ; anti-CD25 PE, Immunotools #22150254; anti CD71 PE, BD #553267; anti-Ter119 FITC, Biolegend #116205; anti-B220 FITC, Immunotools #22159453; anti-IgM PerCP, Santa Cruz #sc-45105) in PBS + 2% FBS for 25 minutes at 4°C. Cells were then washed with PBS and analyzed using a Gallios flow cytometer (Beckman Coulter, Inc., Fullerton, CA). 4',6-diamidino-2-phenylindole (DAPI) was added to the samples in order to discard the dead cells. Changes in the mitochondrial mass, membrane potential and cellular ROS were determined by incubating the cells for 30 min at 37°C with mitoTracker-green, mitoTracker-red or dihydrorhodamine-123, respectively, and analyzed by flow cytometry. The mean fluorescence intensity (MFI) was represented. Fluorescence was collected on the logarithmic scale. The cell population was selected by gating in a forward scatter vs. side scatter dot plot, excluding aggregates and cell debris, and by excluding the DAPI-positive population.

### Cell cycle analysis

In order to determine the cell cycle of irradiated thymocytes, 1*10^6^ cells were fixed in 70% ethanol and stored at -20°C. Samples were washed once with PBS and incubated in 0.1% triton X100, 0.02% RNAse A and 0.0005% propidium iodide (PI) in PBS for 30 minutes at room temperature in the dark. Samples were analyzed in a Coulter XL cytometer and a subsequent cell cycle profile analysis was performed using cytomation summit software.

For cell cycle analysis of thymocyte subsets, 1*10^6^ cells were fixed in 1% paraformaldehyde, and stained with anti-CD4 and anti-CD8 antibodies for 30 minutes at 4°C. Cells were then washed with PBS and incubated for 30 minutes at room temperature with Hoescht 33342 in the dark. Samples were analyzed in a Gallios flow cytometer and cell cycle profile was analyzed with the Watson Pragmatic model.

### Apoptosis assay

Apoptosis was assessed in thymocytes by incubation with annexin V FITC (diluted 1:20, Immunotools) for 15 minutes. Then, PI was added and cells were analyzed by flow cytometry. Samples were analyzed using a Gallios flow cytometer.

### Statistics

Data are represented as the mean and standard deviation (S.D.) of biological replicates. Statistical analysis was performed in GraphPad Prism (La Jolla, CA, USA). One-way Analysis of variance with Bonferroni post-test was used for multiple comparisons. Differences were considered significant when *p < 0.05, **p < 0.01, ***p < 0.001; n.s. not significant.

## SUPPLEMENTARY MATERIALS FIGURES AND TABLES


