# Supplementary material for: Absence of ERK5/MAPK7 delays tumorigenesis in Atm−/− mice
Source: Oncotarget. 2016 Oct 25;7(46):74435–47. doi: 10.18632/oncotarget.12908 (PMC5342677; doi:10.18632/oncotarget.12908)
Supplement: Supplementary file 1 [file oncotarget-07-74435-s001.pdf]

Absence of ERK5/MAPK7 delays tumorigenesis in *Atm*$^{-/-}$ mice

Supplementary Material

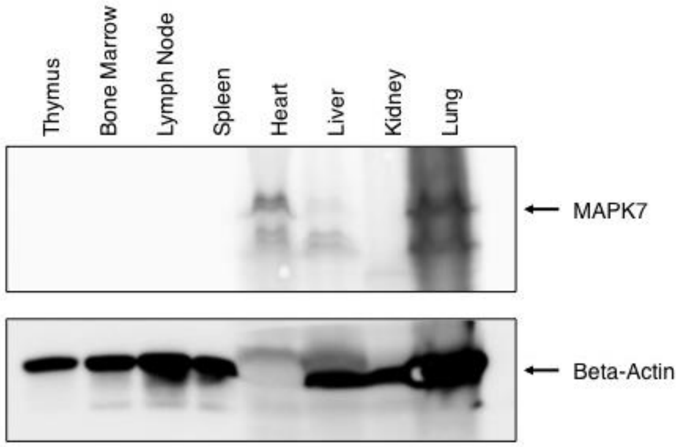

Supplementary Figure 1

MAPK7 expression in tissues of *Mapk7*$^{hemat-/-}$ mice as analyzed by WB. MAPK7 is detected only in non-hematopoietic organs. Beta-Actin was used to normalize sample loading.

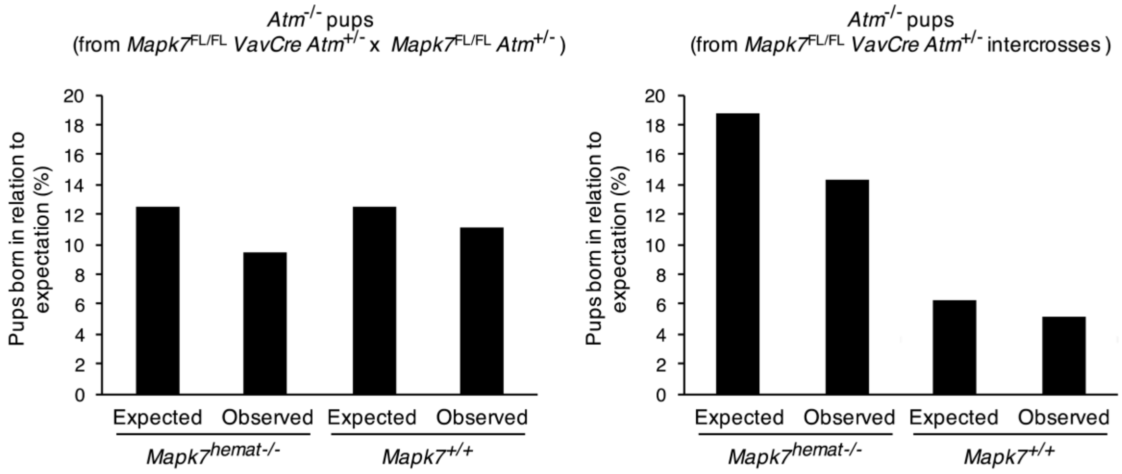

Supplementary Figure 2

Percentage of healthy mice born from (Mapk7$^{\text{hemat-/-}}$*Atm*$^{+/-}$) x (Mapk7$^{loxP/loxP}$*Atm*$^{+/-}$) crosses (n= 244) (**Left**), or from *Mapk7*$^{\text{hemat-/-}}$*Atm*$^{+/-}$ intercrosses (n= 231) (**Right**). p = 0.024 for total *Mapk7*$^{\text{hemat-/-}}$*Atm*$^{-/-}$ mice born as compared with the expected Mendelian frequency.

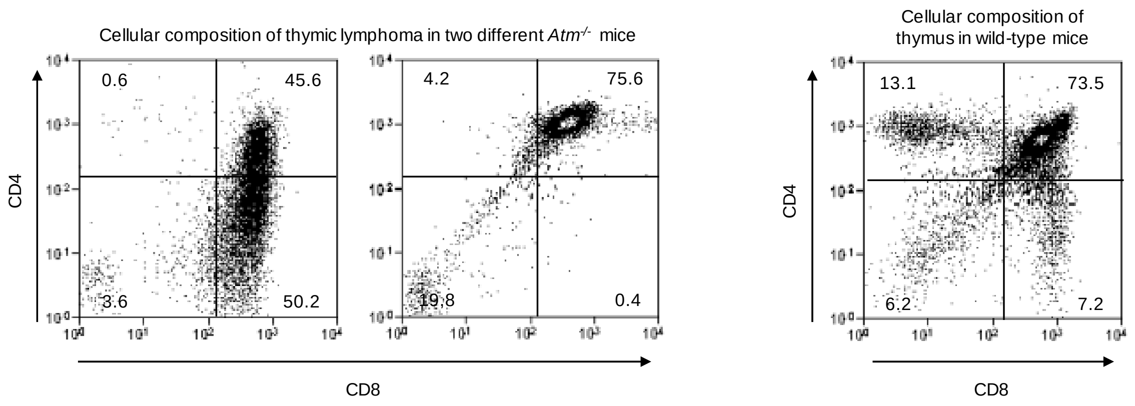

Supplementary Figure 3

Phenotype of thymic cell tumors.

(**Left**) Mice having lost 15 to 20% of their weight were sacrificed, their overgrown thymuses were mechanically disaggregated and the cells were then stained with anti-CD4-APC and anti-CD8-FITC and analyzed by flow cytometry. Two representative samples are shown.

(**Right**) Thymic cells from a 5-week-old wild-type mouse.

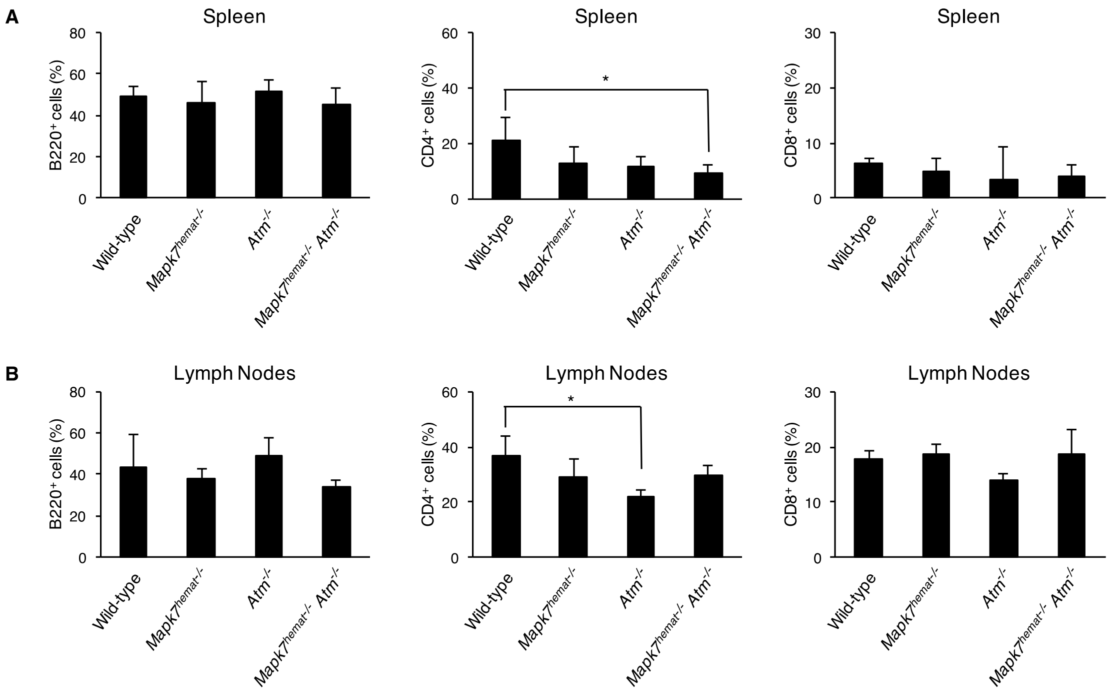

Supplementary Figure 4

Analysis of the distribution of B cells (B220$^+$ cells), CD4$^+$ T cells and CD8$^+$ T cells in spleen (**A**) and superficial cervical lymph nodes (**B**) of mice of the indicated genotypes. Lymphocytes were isolated from 4- and 5-week-old wild-type (n=3), *Mapk7*$^{\text{hemat-/-}}$ (n=7), A*tm*$^{-/-}$ (n=3) and *Mapk7*$^{\text{hemat-/-}}$*Atm*$^{-/-}$ mice (n=6) mice. Data represent mean +/- SD.

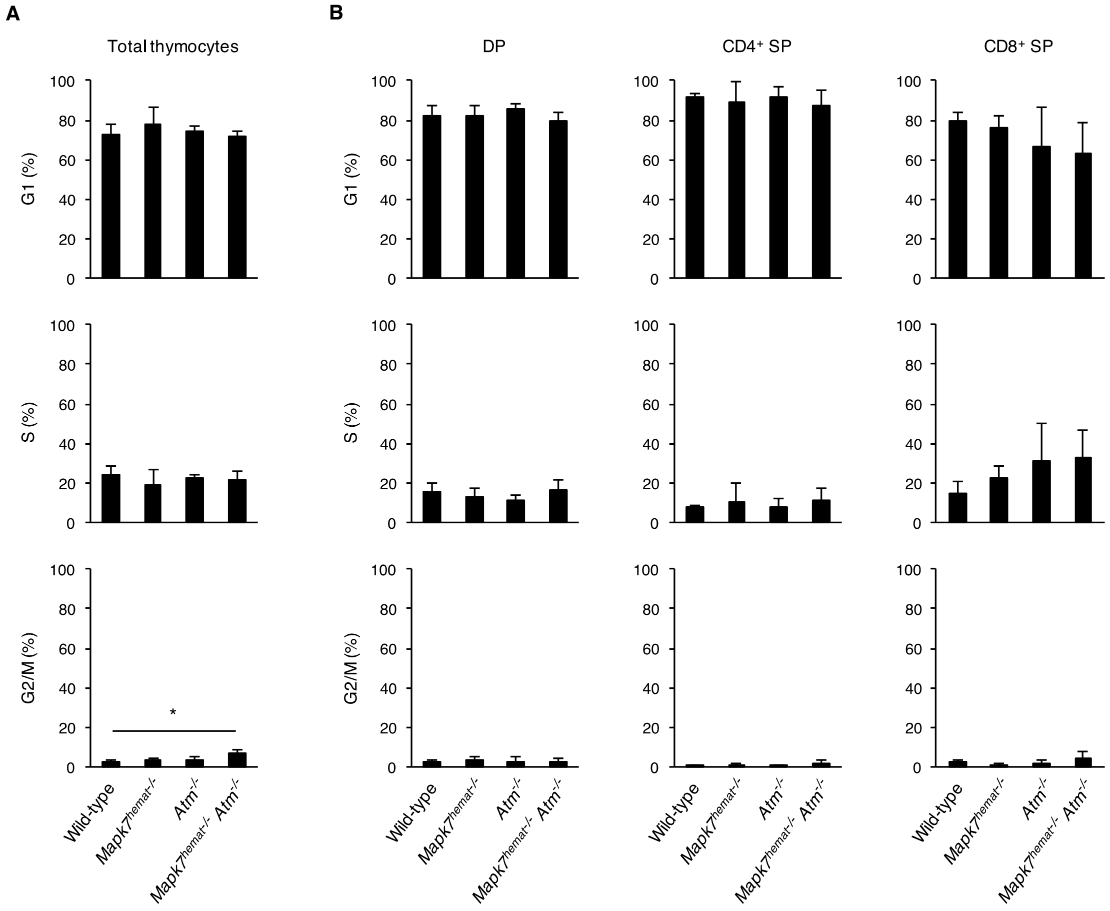

Supplementary Figure 5

Percentage of total thymocytes (**A**) and percentage of DP, SP CD4$^+$ and SP CD8$^+$ thymocyte subsets (**B**) in each cell cycle phase. Thymocytes were fixed in 1% paraformaldehyde, labeled with anti-CD4 and anti-CD8 antibodies as indicated in Fig. 3 and stained with Hoechst 33342.

A.

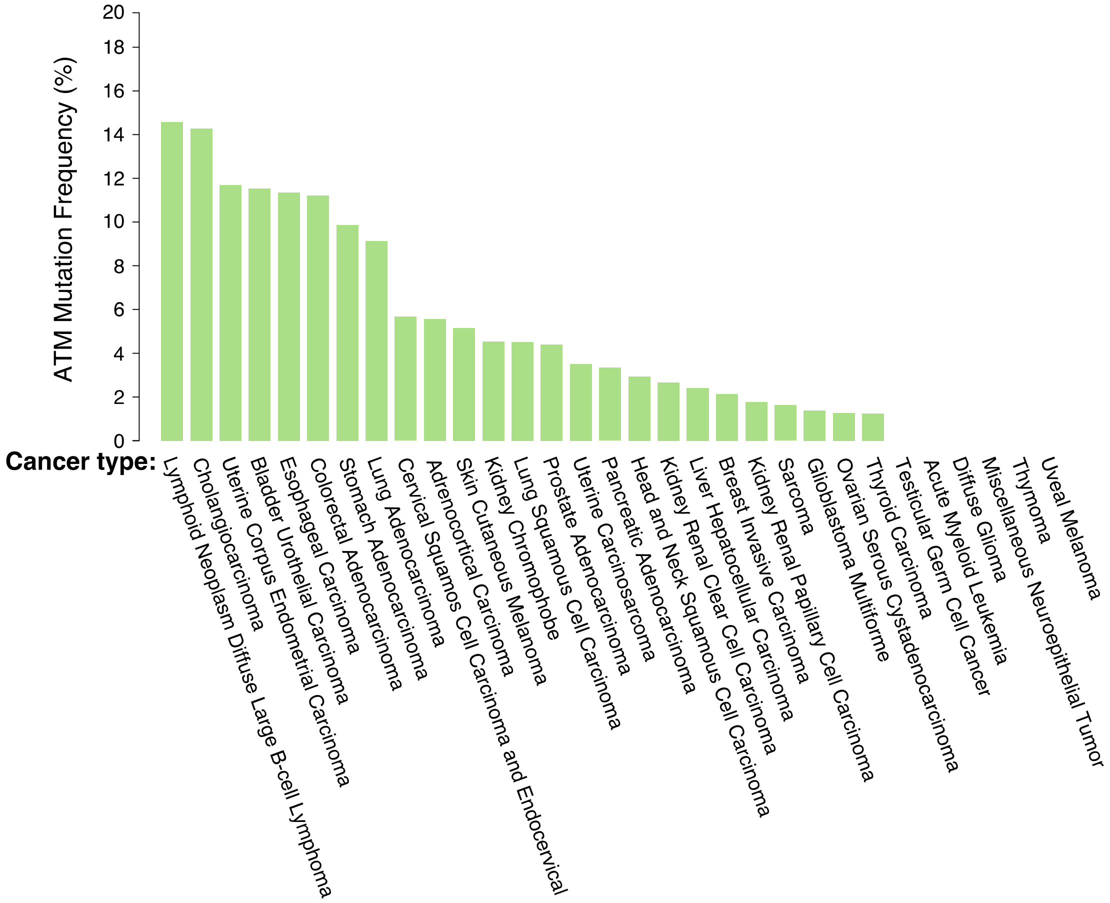

ATM Mutation Frequency (%)
20
18
16
14
12
10
8
6
4
2
0
Cancer type:
Lymphoid Neoplasm Diffuse Large B-cell Lymphoma
Cholangiocarcinoma
Uterine Corpus Endometrial Carcinoma
Bladder Urothelial Carcinoma
Esophageal Carcinoma
Colorectal Adenocarcinoma
Stomach Adenocarcinoma
Lung Adenocarcinoma
Cervical Squamos Cell Carcinoma and Endocervical
Adrenocortical Carcinoma
Skin Cutaneous Melanoma
Kidney Chromophobe
Lung Squamous Cell Carcinoma
Prostate Adenocarcinoma
Uterine Carcinosarcoma
Pancreatic Adenocarcinoma
Head and Neck Squamous Cell Carcinoma
Kidney Renal Clear Cell Carcinoma
Liver Hepatocellular Carcinoma
Breast Invasive Carcinoma
Kidney Renal Papillary Cell Carcinoma
Sarcoma
Glioblastoma Multiforme
Ovarian Serous Cystadenocarcinoma
Thyroid Carcinoma
Testicular Germ Cell Cancer
Acute Myeloid Leukemia
Diffuse Glioma
Miscellaneous Neuroepithelial Tumor
Thymoma
Uveal Melanoma

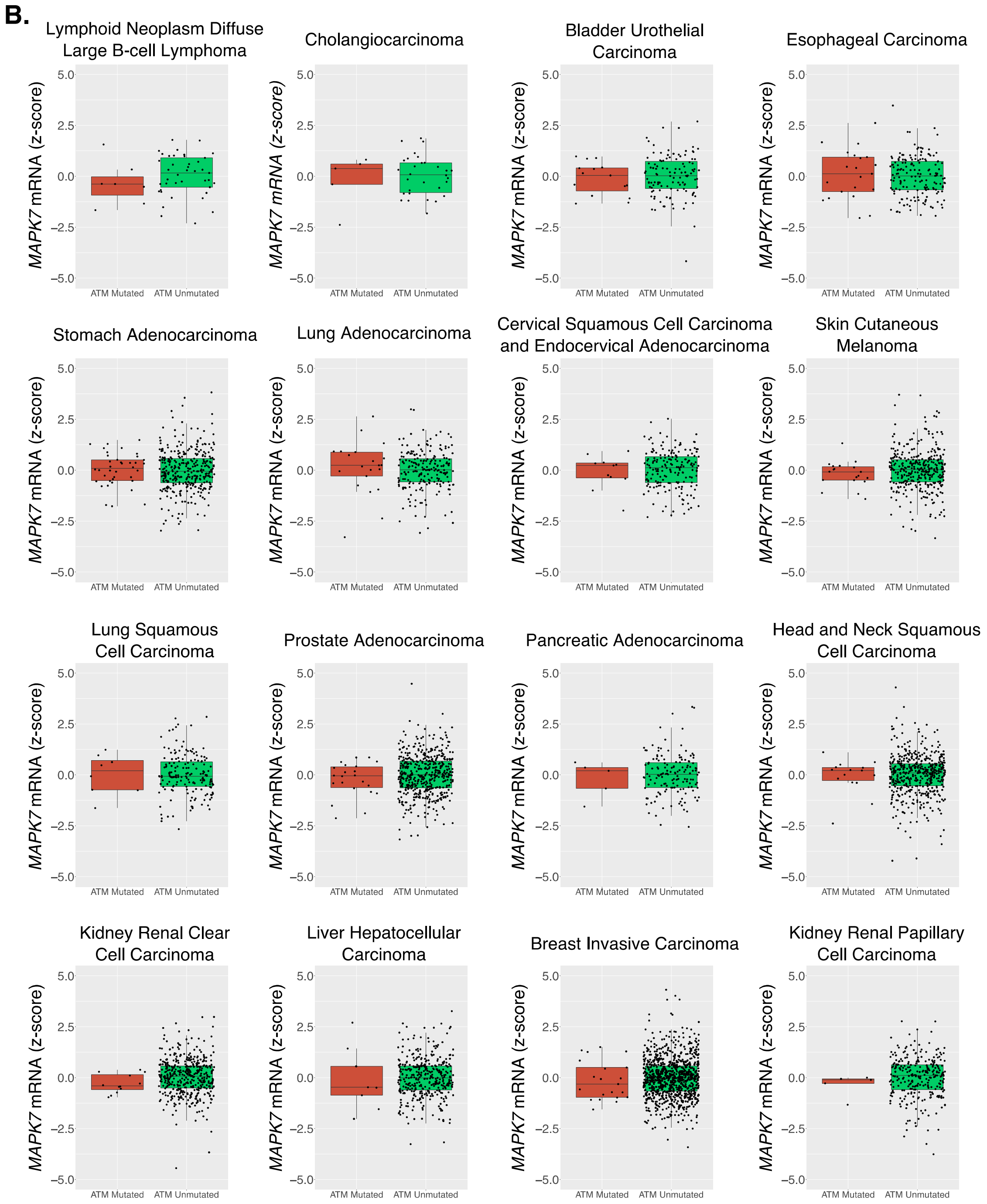

Supplementary Figure 6

(**A**) Analysis of *ATM* mutations in all cancers of the TCGA clinical database shows that ATM is mutated in a broad spectrum of cancers.

(**B**) Z-score plot of *MAPK7* mRNA expression in *ATM* mutated and *ATM* unmutated samples from different human cancer types. Statistical differences in *MAPK7* mRNA expression were not observed between *ATM* mutated and *ATM* unmutated samples.

2. Supplementary Tables

Supplementary Table I. Anatomical characteristics of aging mice.

Only 4 out of 22 *Atm*$^{-/-}$ mice survived longer than 300 days, whereas 11 out of 24 *Mapk7*$^{hemat-/-}$ *Atm*$^{-/-}$ were alive yet. Except for a mouse with ulcerative dermatitis, mice were sacrificed upon losing more than 10% weight. Two mice were not analyzed (----). Histological examination of one *Mapk7*$^{hemat-/-}$*Atm*$^{-/-}$ and two *Atm*$^{-/-}$ mice confirmed an intestinal tumor in one *Atm*$^{-/-}$ mouse, and revealed a normal tissue architecture in spleen, liver, lungs and kidney of the other mice, despite the presence of hepatomegaly and intestinal inflammation in the *Mapk7*$^{hemat-/-}$*Atm*$^{-/-}$ mouse.

Days alive	***Atm***$^{-/-}$ mice	***Mapk7***$^{hemat-/-}$ ***Atm***$^{-/-}$ mice
322		Weight loss and ulcerative dermatitis
351		Hepatoma
370		Hepatomegaly
382	Intestinal tumor	
423		Hepatomegaly and ulcerative dermatitis
429	Hepatomegaly	
438		No observable alterations
447	--------	
452		--------
482		Hepatoma
486	No observable alterations	
488		Thymic lymphoma
526		Ulcerative dermatitis
635		Hepatomegaly and intestinal inflammation
699		Splenomegaly

3. Supplementary Materials and methods

Cancer data analysis

All cancer types in The Cancer Genome Atlas (TCGA) clinical database were queried for mutations in *ATM* using cBio Portal (http://www.cbioportal.org/). Cancer datasets with less than 5 samples showing mutations in *ATM* were excluded. RSEM-normalized mRNA expression levels from RNASeq v2 data of cancers with 5 or more samples showing at least one mutation in *ATM* were downloaded from FireBrowse (http://www.firebrowse.org/; *Broad Institute TCGA Genome Data Analysis Center (2016): Firehose stddata__2016_01_28 run. Broad Institute of MIT and Harvard.* doi:10.7908/C11G0KM9). *MAPK7* mRNA expression for each cancer was selected and $\log_2$ transformed. *ATM* mutated or *ATM* unmutated samples for each cancer were identified and *MAPK7* mRNA expression was z-score transformed for box-plotting. The boxplots were drawn following the Tukey method. Additionally, *MAPK7* mRNA expression for each cancer sample is plotted as an individual point. Differential *MAPK7* mRNA expression was determined using the two-tailed Wilcoxon rank-sum test.
